# Distinct Biomarker Patterns Reveal Metabolic–Inflammatory Profiles Across Mental Disorders

**DOI:** 10.3390/biom16020260

**Published:** 2026-02-05

**Authors:** Krissia F. Godoy, Joice M. A. Rodolpho, Jaqueline Bianchi, Bruna D. L. Fragelli, Fernanda O. Duarte, Luciana Camillo, Gustavo B. Silva, Juliana A. Prado, Carlos Speglich, Fernanda F. Anibal

**Affiliations:** 1 Laboratory of Inflammation and Infectious Diseases, Federal University of São Carlos (UFSCar), São Carlos 13565-905, SP, Brazil; jaqueline_bianchi@yahoo.com.br (J.B.); brufragelli@gmail.com (B.D.L.F.); fefa.duarte74@gmail.com (F.O.D.); lucianacamillo@gmail.com (L.C.); ffanibal@ufscar.br (F.F.A.); 2 Polytechnic School-Data Science and Artificial Intelligence, Pontifical Catholic University of Campinas (PUC Campinas), Campinas 13087-571, SP, Brazil; gustavosilva.bs06@gmail.com; 3 Department of Medicine, Federal University of São Carlos (UFSCar), São Carlos 13565-905, SP, Brazil; julianaprado@ufscar.br; 4 Leopoldo Américo Miguez de Mello Research Center CENPES/Petrobras, Rio de Janeiro 21941-915, RJ, Brazil; carlos.speglich@gmail.com

**Keywords:** anxiety, depression, bipolar disorder, biomarkers, inflammation, metabolism

## Abstract

Mental disorders, including anxiety, depression, and bipolar disorder, are frequently associated with metabolic, inflammatory, and behavioral alterations that modulate their clinical expression and increase the risk of physical comorbidities. This cross-sectional study aimed to characterize the profile of inflammatory, metabolic, and cardiac biomarkers in individuals with mental disorders compared to healthy controls, also considering anthropometric and lifestyle indicators. Fifty volunteers were evaluated and distributed into four groups: control, anxiety, depression, and bipolar disorder. All participants completed the Depression, Anxiety, and Stress Scale—21 items (DASS-21) and underwent blood collection for the assessment of inflammatory biomarkers such as C-Reactive Protein and its high-sensitivity detection (CRP/hs-CRP), Interleukins (IL-6, IL-1β) and Tumor Necrosis Factor alpha (TNF-α), metabolic biomarkers (vitamin D, cortisol, and D-dimer), and cardiac biomarkers such as N-terminal pro-B-type Natriuretic Peptide (NT-proBNP), Creatine Kinase—MB (CK-MB), troponin I (cTnI), and myoglobin (Myo). The results showed a significantly higher body mass index (BMI) in clinical groups, particularly in groups with anxiety and depression. Biomarker analyses revealed significant differences in groups with mental disorders. Elevated levels of CRP (*p* = 0.0038), hs-CRP (*p* = 0.0048), and IL-6 (*p* = 0.0030) were identified in the anxiety group, while the depression group was characterized by reduced vitamin D levels (*p* = 0.0302). Individuals with bipolar disorder presented significantly higher levels of CK-MB (*p* = 0.0016), CRP (*p* < 0.0001), IL-6 (*p* = 0.0198), and IL-1β (*p* = 0.0067). It was also observed that most individuals with mental disorders did not engage in physical activity. This inactivity was associated with worse emotional scores, higher systemic inflammation, and vitamin D deficiency. These findings reinforce the existence of an integrated axis between metabolism, inflammation, and behavior, in which excess weight, sedentary lifestyle, and nutritional deficiencies synergistically contribute to the maintenance of psychiatric symptoms and metabolic vulnerability. Integrating biomarkers, BMI, and behavioral factors may aid in identifying clinical subphenotypes and guiding more precise and individualized therapeutic strategies.

## 1. Introduction

Mental disorders are among the leading contributors to the global burden of disability, impacting not only human suffering but also increasing morbidity and premature mortality. According to estimates from the World Health Organization, over 970 million people currently live with a mental disorder, with depression and anxiety being the most prevalent, while bipolar disorder, although less frequent, stands out due to its clinical severity and high socioeconomic impact [1,2].

Depression is currently one of the main causes of psychosocial functionality impairment worldwide, being associated with cognitive deficits and an increased risk of cardiovascular and metabolic diseases [3]. Regarding anxiety, this prevalence is estimated to be around 300 million individuals worldwide, which frequently coexist with other mental and physical conditions, amplifying the clinical and social burden of these disorders [4]. Likewise, individuals diagnosed with bipolar disorder face a higher risk of cardiovascular complications, with studies suggesting that the likelihood of developing such conditions is approximately twice that observed in the general population [5].

These psychiatric disorders rarely occur in isolation from metabolic factors; alcohol consumption, smoking, impaired sleep patterns, and sedentary behavior are highly prevalent and exacerbate both symptom severity and the risk of physical comorbidities [6]. In particular, overweight and obesity emerge as frequent comorbidities, not only as potential consequences of the disorders but also due to their interconnection through biological, immunoinflammatory, and neuroendocrine mechanisms [7]. Studies indicate that visceral adiposity is associated with a persistent state of low-grade inflammation, promoting neurochemical alterations linked to depression, a concept referred to as the “adiposity-induced inflammation hypothesis” [7].

In this context, chronic activation of the hypothalamic–pituitary–adrenal (HPA) axis serves as a connection point between stress, immunoinflammatory responses, and psychiatric vulnerability. Alterations in cortisol levels, as well as in inflammatory mediators and vasoactive peptides, highlight the interdependence between neural networks, hormones, and inflammation [8]. Circulating biomarkers, including C-reactive protein and its high-sensitivity detection (CRP/hs-CRP), interleukins (IL-6, IL-1β), and the cardiac marker N-terminal pro-B-type Natriuretic Peptide (NT-proBNP), emerge as promising indicators at the interface between mental and cardiometabolic health [9].

Studies suggest that metainflammation constitutes a pathophysiological link between metabolic dysfunctions and psychosomatic disorders, modulating neuroimmune pathways involved in the regulation of mood, behavior, and body perception. Chronic activation of pro-inflammatory cytokines, such as IL-6, TNF-α, and IL-1β, associated with metabolic dysfunction and excess adiposity, promotes alterations in neural plasticity, monoaminergic neurotransmission, cerebral insulin resistance, oxidative stress, and epigenetic modifications, impacting gene expression related to stress response and emotional regulation [10,11,12].

A less explored but crucial aspect is the relationship between elevated Body Mass Index (BMI) (overweight/obesity), psychiatric disorders, and biological responses. Excess adipose tissue—particularly visceral fat—acts as an endocrine organ, releasing adipokines (leptin, adiponectin, resistin) and pro-inflammatory cytokines, generating a state of chronic immune activation [13,14]. This chronic inflammatory state may influence neural networks responsible for mood regulation and stress responses, favoring the maintenance and recurrence of depressive and anxious symptoms [15,16].

Physical activity stands out as an intervention capable of interrupting this pathophysiological cycle. Regular exercise promotes systemic anti-inflammatory effects, reducing biomarkers such as CRP, IL-6, and TNF-α (Tumor Necrosis Factor Alpha), as demonstrated in recent meta-analyses [17].

Aerobic, resistance, or combined training in adults with overweight or obesity reduces pro-inflammatory cytokines and increases anti-inflammatory mediators such as IL-10 [18].

Long-term studies indicate that engaging in physical activity for more than 12 weeks maintains the reduction of these biomarkers in a sustained manner [19]. Furthermore, higher physical fitness appears to attenuate the effects of obesity on inflammation, as active individuals exhibit smaller increases in IL-6 and CRP even with elevated BMI [20]. Experimental evidence suggests that IL-6 released by skeletal muscle during exercise, in the form of a myokine, exerts systemic anti-inflammatory effects, differing from IL-6 secreted by adipose tissue, which has a pro-inflammatory profile [20].

By combining psychological assessments with biological markers, instruments such as the Depression, Anxiety, and Stress Scale—21 items (DASS-21) allow correlations to be drawn between symptom severity and individual biological patterns [21].

This integrative approach enables risk stratification and may pave the way for personalized biomedical assertive interventions for these challenging diseases.

Thus, this study aims to contribute to the understanding of the interface between mental health and metabolism by exploring how inflammatory and metabolic biomarkers are distributed across different psychiatric disorders. By integrating biological, anthropometric, and behavioral variables, it seeks to identify specific patterns that reflect distinct pathophysiological mechanisms and potential therapeutic targets. This integrated approach may assist in characterizing clinical–biological patterns and in formulating more precise preventive and interventional strategies grounded in metabolic and inflammatory modulation.

## 2. Experimental Procedures

### 2.1. Study Design, Participants, and Eligibility

A cross-sectional and observational study was conducted with a total of 50 volunteers, distributed into four groups: healthy controls (*n* = 15), individuals with anxiety (F41, *n* = 11), depression (F32, *n* = 12), and bipolar disorder (F31, *n* = 12). Clinical diagnoses for participants in the psychiatric groups were performed by a psychiatrist, according to the criteria established in the Diagnostic and Statistical Manual of Mental Disorders—DSM-5 and the 10th edition of the International Classification of Diseases (ICD-10).

For volunteers in the mental health group, it was only necessary that they had one ICD code for psychiatric disorders and that they did not have any other health conditions. Exclusion criteria included the presence of autoimmune diseases, diabetes mellitus, systemic arterial hypertension, active infections, or severe chronic illnesses unrelated to the scope of the study.

Control group participants were selected based on the following criteria: body weight within the ideal range, regular physical activity, good sleep quality, no smoking or alcohol consumption, and absence of psychiatric diagnoses or associated clinical comorbidities.

Participants were recruited through convenience sampling as volunteers from psychiatric hospitals, Psychosocial Care Centers (CAPS), and from the ambulatory care of Federal University of São Carlos (UFSCar—São Paulo, Brazil).

All participants signed the Informed Consent Form, approved by the Research Ethics Committee of the Federal University of São Carlos (UFSCar) (protocol nº 54381621.0.0000.5504), registered on Plataforma Brasil, the national unified database for human research, part of the CEP/Conep system, in accordance with the Declaration of Helsinki.

### 2.2. Instruments

All participants completed a questionnaire, which included items related to personal data, physical health, and mental health. Information collected included sex, age (years), weight (kg), height (cm), BMI (kg/m^2^), alcohol consumption, smoking status, physical activity, and sleep quality.

The DASS-21 was used to characterize and assess symptoms of depression, anxiety, and stress. This scale consists of 21 items, with 7 items for each condition. The instrument follows the tripartite model for the clinical assessment of depression, anxiety, and stress, comprising two specific factors (depression and anxiety) and one overlapping factor, called the “stress factor.” The latter is distinct from the other factors but conceptually related due to the close association between depression, anxiety, and stress [21].

Participants indicated the frequency with which they experienced each symptom over the previous week using a 4-point Likert scale, ranging from 0 (did not apply to me) to 3 (applied to me most of the time). Total scores were calculated by summing the individual item scores, reflecting the overall intensity of the symptoms assessed. All psychometric instruments used in this study were translated and validated for the Brazilian population [21].

The questionnaire and blood collection were conducted in person at psychiatric institutions, Psychosocial Care Centers (CAPS), and the Federal University of São Carlos, where a mobile laboratory was set up.

### 2.3. Characteristics of the Studied Volunteers and Samples

To characterize the sample, the following variables were collected: sex (male, female, or other), age, weight, and height, which were used to calculate BMI and its respective classification: underweight (<18.5), normal weight (18.6–24.99), overweight (25–29.99), and obesity (>35). Sleep quality (self-reported as good or poor), physical activity level (active or inactive), tobacco use (current smoker or non-smoker), and alcohol consumption patterns (current consumer or non-consumer) were also assessed.

Blood samples were collected via venipuncture in a single session using vacuum tubes containing ethylenediaminetetraacetic acid (EDTA). The samples were used to analyze the following biomarkers: D-dimer, N-terminal pro B-type natriuretic peptide (NT-proBNP), cortisol, vitamin D, cardiac profile (myoglobin, troponin I, and CK-MB), C-reactive protein (CRP) and high-sensitivity C-reactive protein (hs-CRP), as well as inflammatory cytokines including interleukin 6 (IL-6), interleukin 1β (IL-1β), interleukin 8 (IL-8), and tumor necrosis factor alpha (TNF-α).

Biomarker quantifications were performed by immunofluorescence using specific commercial kits (Celer—Guangzhou Wondfo Biotech Co., Ltd., Guangzhou, China and In Vitro—In Vitro Diagnostica Ltd., Itabira, Brazil), following the manufacturers’ recommended protocols. Whole-blood analyses were conducted using portable equipment (Finecare—Celer—Guangzhou Wondfo Biotech Co., Ltd., Guangzhou, China and QuickSTAR—In Vitro—In Vitro Diagnostica Ltd., Itabira, Brazil). For the quantification of inflammatory cytokines, each participant’s serum was analyzed using a chemiluminescence method on the IMMULITE 1000 system (Siemens—Munich, Germany).

### 2.4. Statistical Analyses

Statistical analyses were performed using GraphPad Prism 9.0 and Python 3.12 (with the pandas, scikit-learn, and seaborn libraries). The normality of variables was assessed using the Shapiro–Wilk test. Continuous variables with a normal distribution were expressed as mean ± standard deviation and compared using a one-way ANOVA. For non-parametric data, the Kruskal–Wallis test was used, followed by Dunn’s post-hoc test. Categorical variables were presented as percentages and compared using the chi-square (χ^2^) test. The significance level for all analyses was set at *p* < 0.05.

The discriminatory capacity of biomarkers was evaluated using Receiver Operating Characteristic (ROC) curves, with the area under the curve (AUC) calculated. An AUC value > 0.7 was considered indicative of good accuracy. Correlations between biomarkers and DASS-21 scores were analyzed using Spearman’s rank correlation coefficient (ρ).

To investigate the predictive power of the biomarkers, two machine learning approaches were employed: Random Forest (RF)—used for an exploratory analysis to identify the relative importance of biomarkers in predicting depression, anxiety, and stress scores (DASS-21) within each clinical group; and logistic regression—employed to analyze the association between biomarkers and the practice of physical activity (a binary outcome: active vs. inactive), identifying factors that are positively or negatively associated with physical activity.

### 2.5. Data Preparation and Preprocessing

Before modeling, the data underwent a rigorous preprocessing procedure. For all machine learning analyses, only individuals with complete data for all 13 biomarkers and the target variable of interest were included, ensuring dataset integrity. The categorical target variable (e.g., ‘mild’, ‘moderate’) was converted into a numeric format using Label Encoding.

The preprocessing of biomarkers was performed distinctly for each model according to its technical requirements. For the Random Forest Model: The biomarker data was used without normalization or standardization. As a tree-based method, Random Forest is not sensitive to the scale of the features, which allows the original data distribution to be preserved for the feature importance analysis.

### 2.6. Random Forest

The Random Forest algorithm was applied to identify the most relevant biomarkers for explaining the DASS-21 scores. The model was trained and validated using stratified 5-fold cross-validation (*k* = 5) to ensure the robustness of the results. The importance of each biomarker was calculated based on the mean decrease in Gini impurity across all trees in the model. Variables with a relative importance greater than 10% were considered clinically relevant. While mean accuracy was monitored as a performance metric, the primary focus of this analysis was variable ranking rather than creating a predictive model for new cases.

## 3. Results

*Sociodemographic and Behavioral Characteristics Among Healthy Individuals and Mental Disorder Groups*

A cross-sectional analysis of demographic socioeconomic, anthropometric and behavioral variables was conducted with a total of 50 volunteers, distributed into the following groups: control (*n* = 15), anxiety (*n* = 11), depression (*n* = 12), and bipolar disorder (*n* = 12), as shown in Table 1.

Age and BMI showed significant differences between groups. The depression group had the highest mean age (47.5 ± 13.07 years), whereas the control group had the lowest mean age (28 ± 9.19 years), with significant differences between control vs. anxiety (^&^), control vs. depression (°), and depression vs. bipolar (^#^).

BMI was higher in the mental disorder groups, particularly in the anxiety group (28.41 ± 8.43 kg/m^2^) compared to the control (23.07 ± 2.85 kg/m^2^) and depression (25.67 ± 3.57 kg/m^2^) groups. Regarding obesity categories, the distribution showed statistically significant differences, with mental disorder groups more prevalent in the overweight and obesity categories. Specifically, 45.45% of volunteers with anxiety were classified as obese, and 50% of individuals with depression or bipolar disorder were overweight.

In terms of gender distribution, females predominated in the anxiety (81.8%) and depression (66.6%) groups, whereas the bipolar disorder group was predominantly male (75%). Only 27.3% of individuals with anxiety and 25% of volunteers with depression reported regular physical activity. Poor sleep quality in groups with mental disorders was reported by 45.45% of individuals with anxiety and 50% of volunteers with depression.

Regarding risk behaviors, smoking was more frequent in the bipolar disorder group (66.6%), and alcohol consumption was most prevalent in the depression group (50%). These data highlight the association between mental disorders and modifiable behavioral factors, indicating potential targets for integrated clinical interventions.

To assess emotional symptoms across groups, the levels of anxiety, depression, and stress were measured using the DASS-21 scale in the control, anxiety, depression, and bipolar disorder groups, which were defined according to clinical diagnoses based on ICD-10. All participants in the clinical groups were under pharmacological treatment.

The results of these assessments are detailed in Table 2. The control group reported minimal scores across the three evaluated domains, with no clinically relevant symptoms.

Turning to the clinical groups, in the group diagnosed with anxiety, 54.55% of the participants exhibited symptoms of anxiety and depression according to the DASS-21, with a predominance of severe anxiety (27.27%) and moderate depression (45.45%).

Among individuals diagnosed with depression, 50% presented anxiety symptoms and 50% depressive symptoms, indicating the simultaneous presence of symptoms across emotional domains.

The bipolar disorder group showed lower but still expressive proportions: 41.67% of participants exhibited anxiety symptoms and 25% depressive symptoms, including one severe case.

These findings indicate that, even among individuals with specific psychiatric diagnoses and under treatment, there is a persistence of overlapping emotional symptoms across different disorders.

Table 3 presents the mean values and standard deviations of key biomarkers across the control, anxiety, depression, and bipolar disorder groups. The biomarkers CK-MB, CRP, IL-6, and IL-1β showed statistically significant differences between groups.

CK-MB levels differed significantly among the groups, with significant differences observed between the control and bipolar disorder groups. CRP showed elevated mean levels in the anxiety and bipolar disorder groups compared to controls, with a significant difference between the control and bipolar disorder groups.

Regarding inflammatory markers, IL-6 levels were significantly higher in the anxiety group compared to the control group, while IL-1β showed differences between the anxiety and bipolar disorder groups.

No significant differences were observed for D-dimer, troponin I, myoglobin, NT-proBNP, cortisol, hs-CRP, vitamin D, TNF-α and IL-8 levels among the groups.

An analysis of the proportion of individuals with clinical alterations is presented in Table 4. The percentage of volunteers with altered biomarkers was evaluated across the different groups (control, anxiety, depression, and bipolar disorder) according to the reference values established for each biomarker.

D-dimer showed alterations in individuals from all groups, with the highest proportion observed in the depression group (50%), although no significant differences were found between groups.

For CK-MB, alterations were only detected in individuals with bipolar disorder (16.6%), without significant differences for this group. Regarding other cardiac markers, troponin I showed no alterations in any group, whereas myoglobin was altered in almost all groups, with the highest proportion in the bipolar disorder group. Similarly, cortisol presented the highest alteration rate in the bipolar group.

NT-proBNP was altered in 33.3% of individuals with depression and absent in the other groups, with a statistically significant difference observed for this marker.

The anxiety group showed the highest proportion of elevated CRP at 27.2%, while hs-CRP was altered in nearly the entire anxiety group (90.9%), over half of the depression group (66.6%), and half of the bipolar disorder group (50%).

Vitamin D levels remained below the optimal range across all groups, with higher percentages observed in individuals with depression and bipolar disorder.

Regarding inflammatory markers, IL-6 was altered in only one individual in both the anxiety and bipolar disorder groups, whereas elevated IL-1β levels were observed exclusively in the bipolar disorder group, with significant differences. TNF-α displayed a high proportion of alterations across all groups.

**Figure 1:**
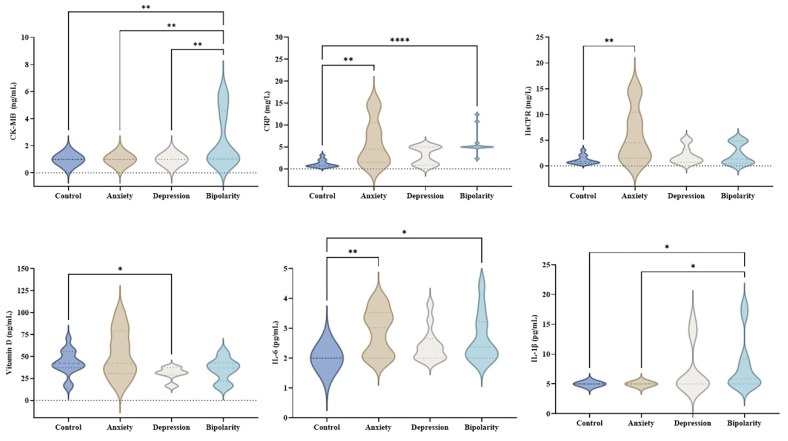
Comparative analysis of biomarker levels among healthy individuals and mental disorder groups. Distribution of CK-MB, CRP, hs-CRP, vitamin D, IL-6, and IL-1β values in control, anxiety, depression, and bipolar disorder groups. Violin plots display the median (dashed line) and data distribution. Statistical analysis was performed using the Kruskal–Wallis test followed by Dunn’s post-hoc test for multiple comparisons. Statistically significant differences are indicated by *p* < 0.05 (*); *p* < 0.01 (**); *p* < 0.0001 (****).

A comparative analysis of biomarkers among the control, anxiety, depression, and bipolar disorder groups revealed significant differences in six markers: CK-MB, CRP, hs-CRP, vitamin D, IL-6, and IL-1β (Figure 1).

Individuals in the bipolar disorder group showed increased CK-MB levels compared with all other groups (control, anxiety, and depression). Elevated CRP and IL-6 levels were also observed in this group compared with the control group, as well as significantly higher IL-1β levels compared with both the control and anxiety groups.

The anxiety group exhibited significantly higher levels of CRP, hs-CRP, and IL-6 only when compared with the control group. Meanwhile, individuals in the depression group presented significantly lower vitamin D levels compared with the control group.

**Figure 2:**
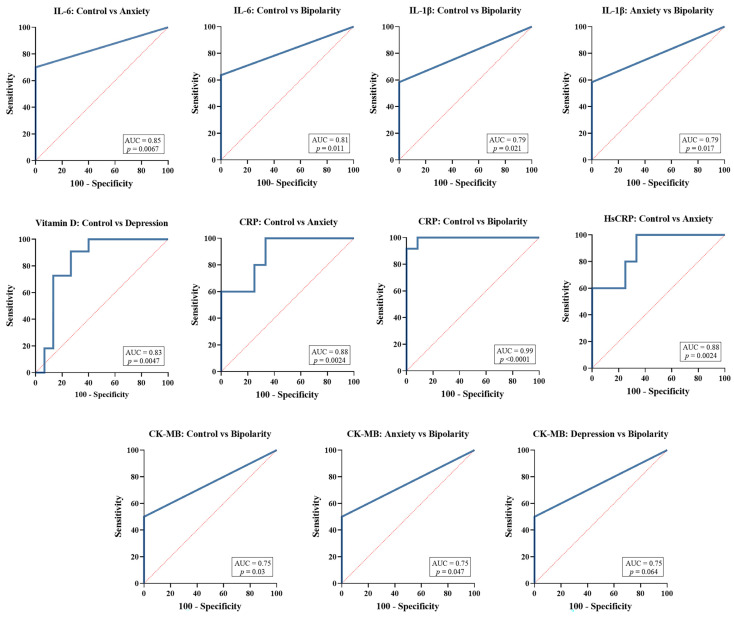
ROC curve analysis of biomarkers showing significant differences between clinical groups. ROC (Receiver Operating Characteristic) curves of biomarkers that demonstrated statistically significant differences in the comparative analysis (Figure 1), evaluating their discriminatory ability between the control, anxiety, depression, and bipolar disorder groups. Performance is expressed by the area under the curve (AUC) and corresponding *p*-values. The blue solid line represents the ROC curve for each biomarker comparison, indicating the diagnostic performance (sensitivity vs. 1–specificity). The red dashed line represents the reference line (line of no discrimination, AUC = 0.5), corresponding to a test with no discriminative ability.

The ROC (Receiver Operating Characteristic) curve analysis (Figure 2) was performed only for the biomarkers and group pairs that exhibited statistically significant differences in the previous comparison (Figure 1).

The IL-6 showed good discriminatory ability between the control and anxiety groups (AUC = 0.85; *p* = 0.0067) and between the control and bipolar disorder groups (AUC = 0.81; *p* = 0.011). The biomarker IL-1β demonstrated relevant discriminatory performance between the control and bipolar groups (AUC = 0.79; *p* = 0.021) and between the anxiety and bipolar groups (AUC = 0.79; *p* = 0.017).

For vitamin D, good accuracy was observed in distinguishing between the control and depression groups (AUC = 0.83; *p* = 0.0047). CRP showed excellent discriminatory capacity between control and anxiety (AUC = 0.88; *p* = 0.0024) as well as between control and bipolar disorder (AUC = 0.99; *p* < 0.0001). Similar results were observed for high-sensitivity CRP (hs-CRP) in the comparison between control and anxiety (AUC = 0.88; *p* = 0.0024).

For CK-MB, the discriminatory power was moderate in comparisons between control and bipolar disorder (AUC = 0.75; *p* = 0.030), anxiety and bipolar disorder (AUC = 0.75; *p* = 0.047), and depression and bipolar disorder (AUC = 0.75; *p* = 0.064).

**Figure 3:**
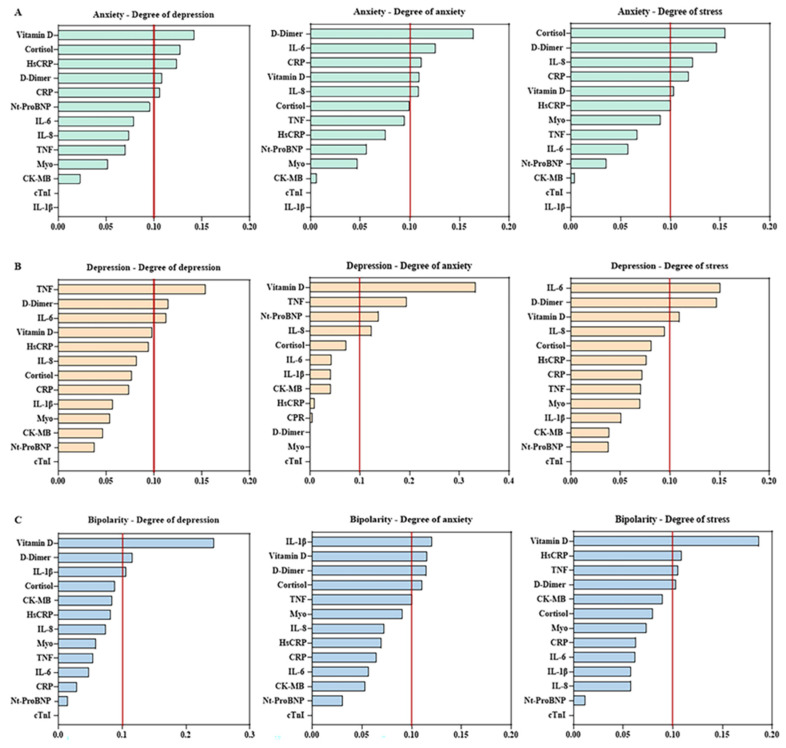
Importance of biomarkers in predicting DASS-21 depression, anxiety, and stress scores in clinical groups with mental disorders. Relative importance analysis of biomarkers in predicting DASS-21 scores for depression, anxiety, and stress in individuals diagnosed with anxiety (**A**), depression (**B**), and bipolar disorder (**C**). The analysis was performed using a machine learning model, and biomarkers with importance values above 0.10 (10%) (red line) were considered the most relevant for each clinical class.

A biomarker importance analysis was conducted based on the DASS-21 scores, as illustrated in Figure 3. The results revealed distinct biomarker profiles associated with anxiety, depression, and stress symptoms across the clinical groups (anxiety, depression, and bipolar disorder).

In the anxiety group (Figure 3A), depression scores showed greater relevance for vitamin D, cortisol, hs-CRP, D-dimer, and CRP. Anxiety severity was mainly predicted by D-dimer, IL-6, CRP, vitamin D and IL-8, whereas stress levels were primarily associated with cortisol, D-dimer, IL-8, CRP, vitamin D, and hs-CRP.

In the depression group (Figure 3B), depression severity showed higher importance for TNF-α, D-dimer, and IL-6. Anxiety levels were more strongly predicted by vitamin D, TNF-α, NT-proBNP, and IL-8, while stress was mainly associated with IL-6, D-dimer, and vitamin D.

Among individuals with bipolar disorder (Figure 3C), depression severity was primarily influenced by vitamin D, D-dimer, and IL-1β. Anxiety levels were associated with IL-1β, vitamin D, D-dimer, and cortisol, whereas stress prediction was driven by vitamin D, hs-CRP, TNF-α, and D-dimer.

**Figure 4:**
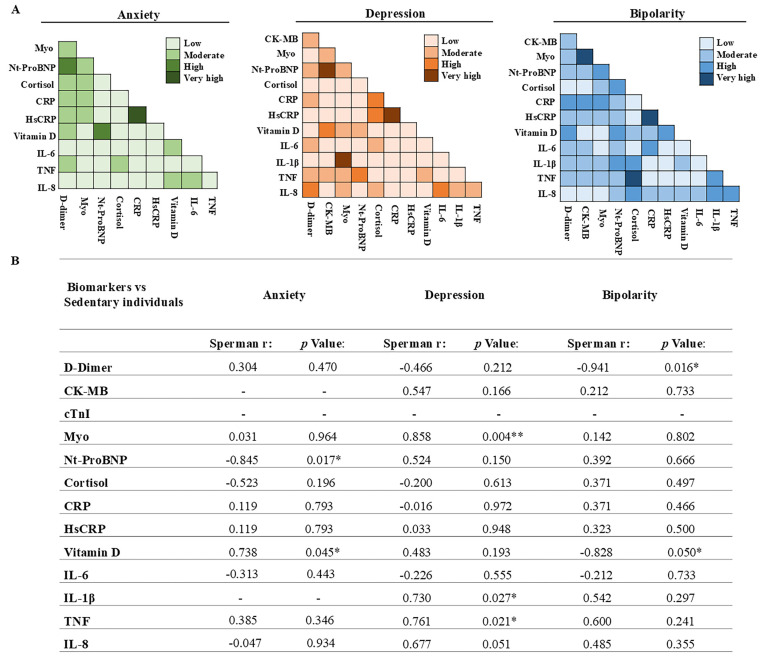
Correlation of biomarkers with psychiatric disorders in individuals who do not engage in physical activity. (**A**) The color scale represents the intensity of the correlations, categorized as low, moderate, high, and very high. (**B**) Spearman correlation analysis between biomarkers and anxiety, depression, and bipolar disorder in sedentary individuals revealed significant associations for NT-proBNP and anxiety, vitamin D and anxiety, myoglobin, IL-1β, and TNF -α with depression, and D-dimer and vitamin D with bipolar disorder (* *p* < 0.05; ** *p* < 0.01).

Figure 4 presents a correlation analysis performed among biomarkers in patients who did not engage in physical activity from the clinical groups with anxiety, depression, and bipolar disorder. The correlations are categorized by color intensity, ranging from low to very high, providing a clear visualization of the strength and pattern of association among biomarkers within each clinical condition (Figure 4A).

In addition, the Spearman correlation (Figure 4B) between biomarkers and psychiatric disorders in sedentary individuals revealed relevant associations. In the anxiety group, a significant negative correlation was observed between NT-proBNP and anxiety (r = −0.845; *p* = 0.017), along with a positive correlation between vitamin D and anxiety (r = 0.738; *p* = 0.045).

In the depression group, strong positive correlations were identified for inflammatory and muscle injury markers, including myoglobin (r = 0.858; *p* = 0.004), IL-1β (r = 0.730; *p* = 0.027), and TNF- α (r = 0.761; *p* = 0.021).

In the bipolar disorder group, significant negative correlations were observed between D-dimer and bipolarity (r = −0.941; *p* = 0.016) and between vitamin D and bipolarity (r = −0.828; *p* = 0.050). Other biomarkers did not show statistically significant correlations.

## 4. Discussion

The present study investigated the metabolic and inflammatory profile of individuals with mental disorders such as anxiety, depression, and bipolar disorder in comparison with healthy controls, integrating clinical variables, serum biomarkers, and behavioral factors such as physical activity. The findings revealed that patients with mental disorders presented higher mean age, elevated BMI levels, and greater prevalence of overweight and obesity. This trend is widely supported by the literature, which identifies obesity as both a risk factor and an aggravating element for psychiatric symptoms, mediated by systemic inflammation and metabolic dysfunction [22,23].

The accumulation of adipose tissue contributes to increased levels of pro-inflammatory cytokines such as IL-6 and TNF-α, which are capable of modulating neural pathways related to mood and behavior, creating a state of low-grade inflammation that promotes fatigue, sedentary behavior, and clinical worsening [24].

In addition to anthropometric and behavioral aspects, a distinct inflammatory pattern was observed among the clinical groups, with significant increases in CRP, hsCRP, IL-6, and IL-1β, along with decreased serum vitamin D levels across all analyzed mental disorders. These findings are consistent with previous studies describing the activation of the inflammatory–immune axis as a central component of psychiatric pathophysiology, particularly in conditions associated with mood and anxiety disorders [22,25].

In particular, IL-6 has been consistently associated with the severity of anxiety symptoms and increased physiological stress, even in individuals without prior psychiatric treatment [25,26]. Elevated levels of this cytokine promote hyperactivity of the hypothalamic–pituitary–adrenal axis, contributing to sleep disturbances, reduced energy, and emotional dysregulation, while low vitamin D levels impair immunomodulatory control and neuronal functionality [27].

Thus, the relationship between chronic inflammation, metabolic dysfunction, and vitamin D deficiency may represent a shared biological mechanism linking sedentary behavior to the persistence of anxious and depressive symptoms observed in this sample.

The binary analysis of physical activity revealed distinct biomarker patterns associated with sedentary behavior in each clinical condition, indicating that physical inactivity is sustained by specific biological mechanisms in anxiety, depression, and bipolar disorder. Among patients with anxiety, higher IL-6 levels were strongly associated with the absence of physical activity, reflecting a diffuse inflammatory profile characterized by activation of the innate immune system and possible resistance to physiological stress. This finding is consistent with studies reporting elevated IL-6 in individuals with anxiety, associated with autonomic nervous system imbalance and increased fatigue [25].

Conversely, in the depressive group, sedentary behavior was linked to elevated CRP, hsCRP, and cortisol, along with vitamin D deficiency, reinforcing the presence of low-grade systemic inflammation and HPA axis dysfunction—patterns widely described in treatment-resistant depression and chronic fatigue states [22,27]. In individuals with bipolar disorder, sedentary behavior was characterized by elevated IL-1β, reduced vitamin D, and increased myoglobin, suggesting activation of the inflammasome (IL-1 pathway) and possible muscular or metabolic impairment secondary to inactivity. These results are in line with recent evidence describing IL-1β as one of the most sensitive cytokines to mood fluctuations and the underlying inflammatory state in bipolar patients [16,28,29].

Importantly, the present findings suggest that sedentary behavior in mental disorders should not be interpreted solely as a behavioral choice, but rather as a biologically sustained state influenced by inflammatory and neuroendocrine dysregulation. Inflammatory cytokines such as IL-6 and IL-1β have been shown to induce sickness behavior, characterized by fatigue, reduced motivation, and decreased physical activity, even in the absence of overt medical illness [30,31].

This mechanism may partly explain the distinct biomarker patterns associated with physical inactivity observed across anxiety, depression, and bipolar disorder in this study.

The association between obesity, inflammation, and cardiovascular risk also emerged as a central component, reinforcing the interconnection between metabolism, sedentary behavior, and mental health. The clinical groups with mental disorders exhibited significantly higher BMI values than the control group, as well as a higher prevalence of overweight and obesity, particularly among individuals with anxiety and depression. These findings are consistent with recent evidence identifying obesity as an independent risk factor for the development and worsening of depressive and anxious symptoms, mediated by chronic metabolic and inflammatory dysfunctions [22,23,32].

Elevated CK-MB and NT-proBNP levels, particularly in patients with bipolar disorder and depression, may reflect subclinical myocardial stress associated with obesity and reduced physical activity, suggesting an overlap between cardiovascular risk and psychiatric vulnerability. Previous studies have demonstrated that NT-proBNP—a traditional biomarker of cardiac dysfunction—can also be elevated in individuals with depression even in the absence of structural heart disease, possibly as a response to systemic physiological and neuroendocrine stress [33,34].

Moreover, the presence of D-dimer elevation across all clinical groups reinforces the hypothesis of systemic procoagulant and inflammatory activation, frequently linked to metabolic inflammation and sedentary behavior [35,36]. This combination of persistent inflammation, endothelial dysfunction, and metabolic imbalance may represent a cardiometabolic risk phenotype in mental disorders, in which biomarkers such as NT-proBNP and CK-MB reflect not only cardiovascular status but also the cumulative impact of physical inactivity, excess weight, and low-grade inflammation [34,36,37].

In summary, the results of this study reinforce that systemic inflammation, vitamin D deficiency, excess weight, and sedentary behavior constitute interdependent biological mechanisms involved in the pathophysiology of mental disorders. The interaction among these factors establishes an unfavorable metabolic state that contributes to the intensification of emotional symptoms and the increased cardiometabolic vulnerability observed in these patients. These findings highlight the importance of an integrated clinical approach that combines psychiatric management with strategies for inflammatory modulation, nutritional correction, and promotion of physical activity, thereby supporting metabolic balance and overall well-being.

An additional limitation relates to the modest sample size. This was mainly due to the strict inclusion criteria adopted in this study, which aimed to recruit individuals with a single, well-defined psychiatric diagnosis (anxiety, depression, or bipolar disorder), without concomitant psychiatric comorbidities or metabolic disorders. This approach was intentionally chosen to minimize clinical and biological heterogeneity and allow for a more precise interpretation of biomarker profiles. However, such a homogeneous clinical presentation is uncommon in psychiatric populations, where comorbidity is highly prevalent, which limited the availability of participants. Consequently, while this strategy strengthens internal validity, it may reduce the sample size and the generalizability of the results.

## 5. Conclusions

Mental disorders reflect an imbalance that extends beyond the psychological domain, involving metabolism, inflammation, and behavior. This study identified relevant and distinct biomarkers among the different clinical conditions, underscoring the importance of an integrated approach that combines psychiatric management with nutritional, anti-inflammatory, and physical interventions. The identification of these biomarkers highlights the importance of personalized strategies capable of promoting better clinical outcomes and reducing the risk of cardiovascular and metabolic comorbidities in patients with mental disorders.

## Figures and Tables

**Table 1:** Comparison of sociodemographic, anthropometric, and behavioral variables among healthy volunteers and individuals with mental disorders. Data are presented as mean ± standard deviation (MD ± SD) for continuous variables and percentages for categorical variables. One-way ANOVA and chi-square tests were used to assess statistically significant differences for continuous and categorical data, respectively. *p*-values listed in the table indicate overall statistical differences between groups, and specific pairwise comparisons were conducted using Dunn’s post-hoc test, as indicated below (* *p* < 0.05; *** *p* < 0.001; **** *p* < 0.0001).

Variables		Overall (*n* = 50)	Control (*n* = 15)	Anxiety (*n* = 11)	Depression (*n* = 12)	Bipolarity (*n* = 12)	*p*-Value
Age (MD/SD):		36 ± 12.81	28 ± 9.19	39.5 ± 14.46	47.5 ± 13.07	35.5 ± 7.94	0.0002 ^&^°^#^
BMI (kg/m^2^) (MD/SD):		24.53 ± 5.46	23.07 ± 2.85	28.41 ± 8.43	25.67 ± 3.57	26.24 ± 3.44	0.0008 ^&γ^
Obesity degree:							0.0004 ***
	Underweight:	2 (4%)	0 (0%)	1 (9.09%)	1 (8.33%)	0 (0%)	
	Ideal weight:	25 (50%)	15 (100%)	2 (18.18%)	4 (33.33%)	4 (33.33)	
	Overweight:	15 (30%)	0 (0%)	3 (27.27%)	6 (50%)	6 (50%)	
	Obesity:	8 (16%)	0 (0%)	5 (45.45%)	1 (8.33%)	2 (16.66%)	
Gender:							0.0253 *
	Masculine:	24 (48%)	9 (60%)	2 (18.18%)	4 (33.33%)	9 (75%)	
	Feminine:	26 (52%)	6 (40%)	9 (81.81%)	8 (66.66%)	3 (25%)	
Physical activity:							0.0002 ***
	No:	23 (46%)	0 (0%)	8 (72.72%)	9 (75%)	6 (50%)	
	Yes:	27 (54%)	15 (100%)	3 (27.27%)	3 (25%)	6 (50%)	
Sleep quality:							0.0183 *
	Good:	35 (70%)	15 (100%)	6 (54.54%)	6 (50%)	8 (66.66%)	
	Bad:	15 (30%)	0 (0%)	5 (45.45%)	6 (50%)	4 (33.33%)	
Smoking:							<0.0001 ****
	Non-Smoker:	39 (78%)	15 (100%)	11 (100%)	9 (75%)	4 (33.33%)	
	Smoker:	11 (22%)	0 (0%)	0 (0%)	3 (25%)	8 (66.66%)	
Alcoholic beverages:							0.0178 *
	Non-drinker:	39 (78%)	15 (100%)	8 (72.72%)	6 (50%)	10 (83.33%)	
	Drinker	11 (22%)	0 (0%)	3 (27.27%)	6 (50%)	2 (16.66%)	

BMI—Body Mass Index. ^&^ *p* < 0.05 Control vs. Anxiety. ° *p* < 0.05 Control vs. Depression. ^#^ *p* < 0.05 Depression vs. Bipolarity. ^γ^ *p* < 0.05 Anxiety vs. Depression.

**Table 2:** Classification of anxiety, depression, and stress scores based on the DASS-21 scale. Distribution and comparison of anxiety, depression, and stress levels among healthy individuals and those with mental disorders. Data are presented as percentages. One-way ANOVA and chi-square tests were used to evaluate statistically significant differences (*p* < 0.05).

		Control (*n* = 15)	Anxiety (*n* = 11)	Depression (*n* = 12)	Bipolarity (*n* = 12)	*p*-Value
**Anxiety**						0.0705
	No anxiety	15 (100%)	5 (45.45%)	6 (50%)	7 (58.33%)	
	Mild	0 (0%)	1 (9.09%)	0 (0%)	2 (16.66%)	
	Moderate	0 (0%)	2 (18.18%)	3 (25%)	1 (8.33%)	
	Severe	0 (0%)	3 (27.27%)	3 (25%)	2 (16.66%)	
**Depression**						0.0881
	No depression	14 (93.33%)	5 (45.45%)	6 (50%)	9 (75%)	
	Mild	1 (6.66%)	1 (9.09%)	2 (16.66%)	0 (0%)	
	Moderate	0 (0%)	5 (45.45%)	4 (33.33%)	2 (16.66%)	
	Severe	0 (0%)	0 (0%)	0 (0%)	1 (8.33%)	
**Stress**						0.2778
	No stress	15 (100%)	7 (63.63%)	8 (66.66%)	10 (83.33%)	
	Mild	0 (0%)	3 (27.27%)	3 (25%)	1 (8.33%)	
	Moderate	0 (0%)	1 (9.09%)	1 (8.33%)	1 (8.33%)	
	Severe	0 (0%)	0 (0%)	0 (0%)	0 (0%)	

**Table 3:** Comparison of mean biomarker levels between healthy individuals and mental disorder groups. Absolute biomarker values are presented by group as mean ± standard deviation (MD ± SD). Global comparisons among the groups (control, anxiety, depression, and bipolar disorder) were performed using one-way ANOVA, with a significance level of *p* < 0.05. *p*-values listed in the table indicate overall statistical differences among groups, and specific pairwise comparisons were conducted using Dunn’s post-hoc test, as indicated below.

	Control (*n* = 15) (MD/SD)	Anxiety (*n* = 11) (MD/SD)	Depression (*n* = 12) (MD/SD)	Bipolarity (*n* = 12) (MD/SD)	*p*-Value
**D-Dimer**	0.18 ± 0.88	0.25 ± 0.38	0.38 ± 0.53	0.18 ± 0.43	0.7980
**CK-MB**	1 ± 0.10	1 ± 0.15	1 ± 0.66	1.09 ± 2.01	0.0325 °
**cTnI**	0.1 ± 0	0.1 ± 0	0.1 ± 0	0.1 ± 0	-
**Myo**	10 ± 99.23	10 ± 14.11	10.21 ± 18.42	16.77 ± 47.19	0.1474
**Nt-ProBNP**	100 ± 0	100 ± 65.96	100 ± 194.13	100 ± 35.79	0.1951
**Cortisol**	420.22 ± 174.27	455.23 ± 121.24	375.61 ± 225.85	324.55 ± 285.91	0.7755
**CRP**	0.92 ± 4.59	4.97 ± 9.02	4.08 ± 5.26	5 ± 2.78	0.0136 °
**hsCRP**	0.92 ± 4.59	4.97 ± 9.02	1.5 ± 5.43	1.3 ± 1.99	0.0610
**Vitamin D**	42.3 ± 14.25	41.65 ± 23	31.85 ± 21.35	36.95 ± 13.01	0.0760
**IL-6**	2 ± 0.58	3.27 ± 6.28	2.16 ± 0.62	2.37 ± 1.7	0.0299 ^&^
**IL-1β**	5 ± 6.73	5 ± 0	5 ± 4.24	5.85 ± 4.63	0.0497 ^#^
**TNF**	9.91 ± 5.03	10.7 ± 2.88	10 ± 4.42	8.32 ± 4.84	0.7092
**IL-8**	9.76 ± 6.13	14.8 ± 16.17	10.4 ± 5.52	8.77 ± 4.94	0.4283

° *p* < 0.05 Control vs. Bipolarity. ^&^ *p* < 0.05 Control vs. Anxiety. ^#^ *p* < 0.05 Anxiety vs. Bipolarity.

**Table 4:** Proportion of individuals with altered biomarkers in healthy volunteers and mental disorder groups. Analysis of the percentage of individuals with values outside the reference range for each biomarker in healthy and mental disorder groups. Reference values are indicated in the second column, and data are expressed as the percentage of individuals with clinical alterations relative to the total of each group. The chi-square test was used to evaluate statistically significant differences (* *p* < 0.05; ** *p* < 0.01).

Biomarkers	Reference Values	Control (*n* = 15)	Anxiety (*n* = 11)	Depression (*n* = 12)	Bipolarity (*n* = 12)	*p*-Value
**D—Dimer**	(0–0.5) mg/L	7 (46.6%)	3 (27.2%)	6 (50%)	2 (16.6%)	0.2538
**CK-MB**	(0–5) ng/mL	0 (0%)	0 (0%)	0 (0%)	2 (16.6%)	0.0859
**cTnI**	(0–0.3) ng/mL	0 (0%)	0 (0%)	0 (0%)	0 (0%)	-
**Myo**	(0–58) ng/mL	1 (6.6%)	0 (0%)	1 (8.3%)	3 (25%)	0.2173
**Nt-ProBNP**	(0–300 < 75 years 0–450 > 75 years-pg/mL)	0 (0%)	0 (0%)	4 (33.3%)	0 (0%)	0.0032 **
**Cortisol**	(201.31–536.54 nmol/L)	4 (26.6%)	0 (0%)	3 (25%)	4 (33.3%)	0.2353
**CRP**	(0–10) mg/L	1 (6.6%)	3 (27.2%)	1 (8.3%)	2 (16.6%)	0.4453
**hsCRP**	(0–1) mg/L	7 (46.6%)	10 (90.9%)	8 (66.6%)	6 (50%)	0.1004
**Vitamin D**	>30 ng/mL	2 (13.3%)	2 (18.1%)	3 (25%)	3 (25%)	0.8460
**IL-6**	(5.9 pg/mL)	0 (0%)	1 (9%)	0 (0%)	1 (8.3%)	0.4831
**IL-1B**	(5 pg/mL)	5 (33.3%)	0 (0%)	4 (33.3%)	7 (58.3%)	0.0291 *
**TNF**	(8.1 pg/mL)	14 (93.3%)	9 (81.8%)	9 (75%)	7 (58.3%)	0.1780
**IL-8**	(62 pg/mL)	0 (0%)	0 (0%)	0 (0%)	0 (0%)	-

## Data Availability

The original contributions presented in this study are included in the article. Further inquiries can be directed to the corresponding authors.
